# Supplementary material for: Distinct molecular cues ensure a robust microtubule-dependent nuclear positioning in the Drosophila oocyte
Source: Nat Commun. 2017 Apr 27;8:15168. doi: 10.1038/ncomms15168 (PMC5414183; doi:10.1038/ncomms15168)
Supplement: Supplementary Information — Supplementary Figures [file ncomms15168-s1.pdf]

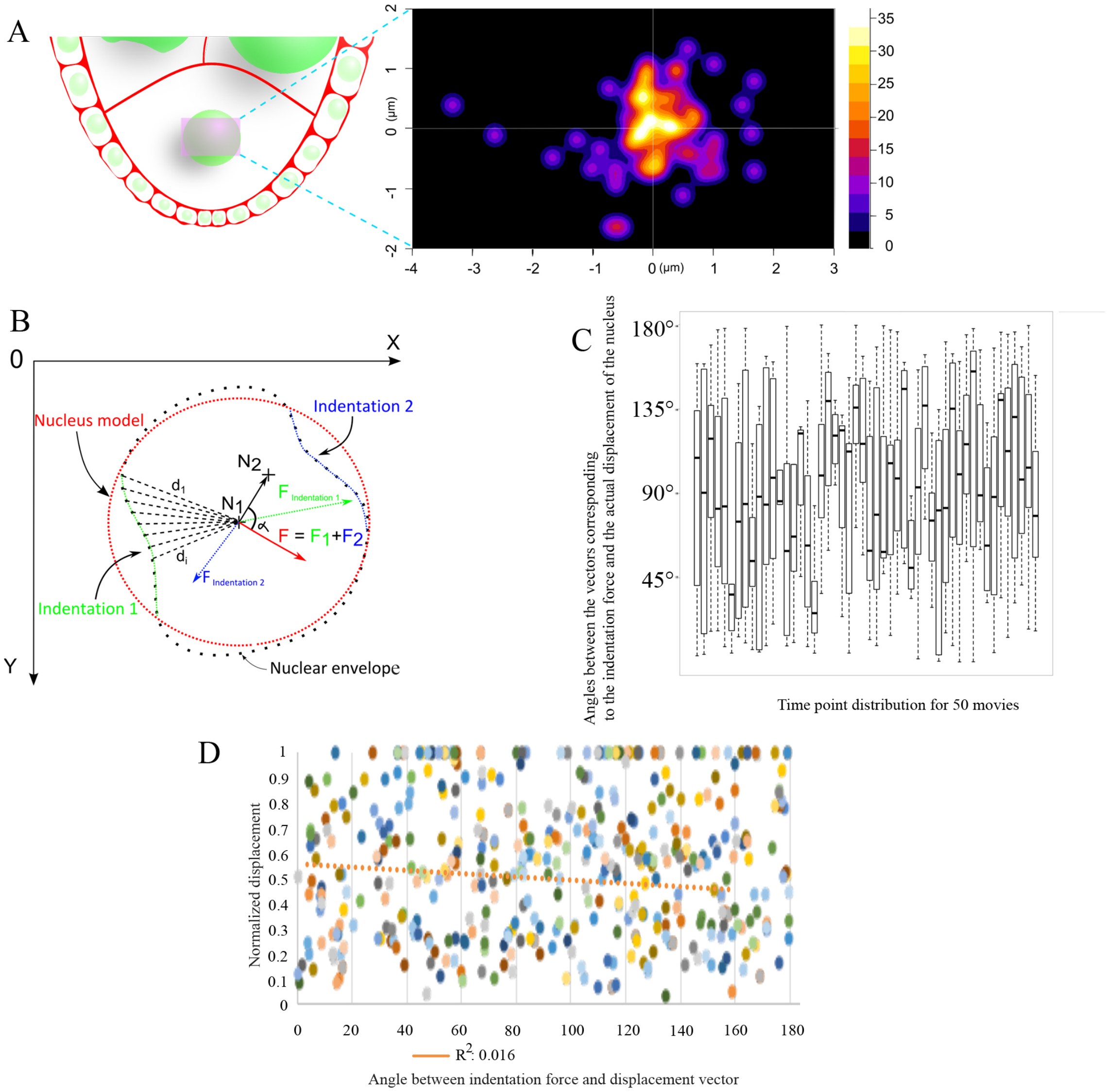

Supplementary Figure 1: Relation between directionality and indentation.

Heat map representation illustrating the density of the various positions of the nucleus prior to migration in a zone of 21 μm^2. The various positions of the nuclear center of mass is considered. (B) Measurement principle of the angle α between the resulting indentation force $\vec{F}$ and the displacement vector $\overrightarrow{N1N2}$ for the nucleus. N_1 and N_2 correspond to the nuclear position at t_1 and t_2. $\vec{F}$ is the sum of $\vec{F}_{indentation1} + \vec{F}_{indentation2}$ which are the forces creating the indentations 1 (green) and 2 (blue). The black dotted line represents the nuclear envelope at t_1. The red dotted circle represents the nuclear envelope model without indentations. Its radius is

equal to the average radius of the black dotted line. Indentations are the green and blue portions of the black dotted line with a radius smaller than that of the circular envelope (red dotted line). α is the angle between $\overrightarrow{N1N2}$ and $\vec{F}$. (C) Box plot showing the distribution of angles between the vectors corresponding to the indentation force and the actual displacement of the nucleus for each of the 50 movies analysed in this study (n=429). (D) Normalised displacement of the nuclei with respect to the angle between the force vector corresponding to the nuclear indentation and the nuclear displacement vector. The correlation coefficient R^2 is 0.016 with the correlation function y = - 0.0006x + 0.5564.

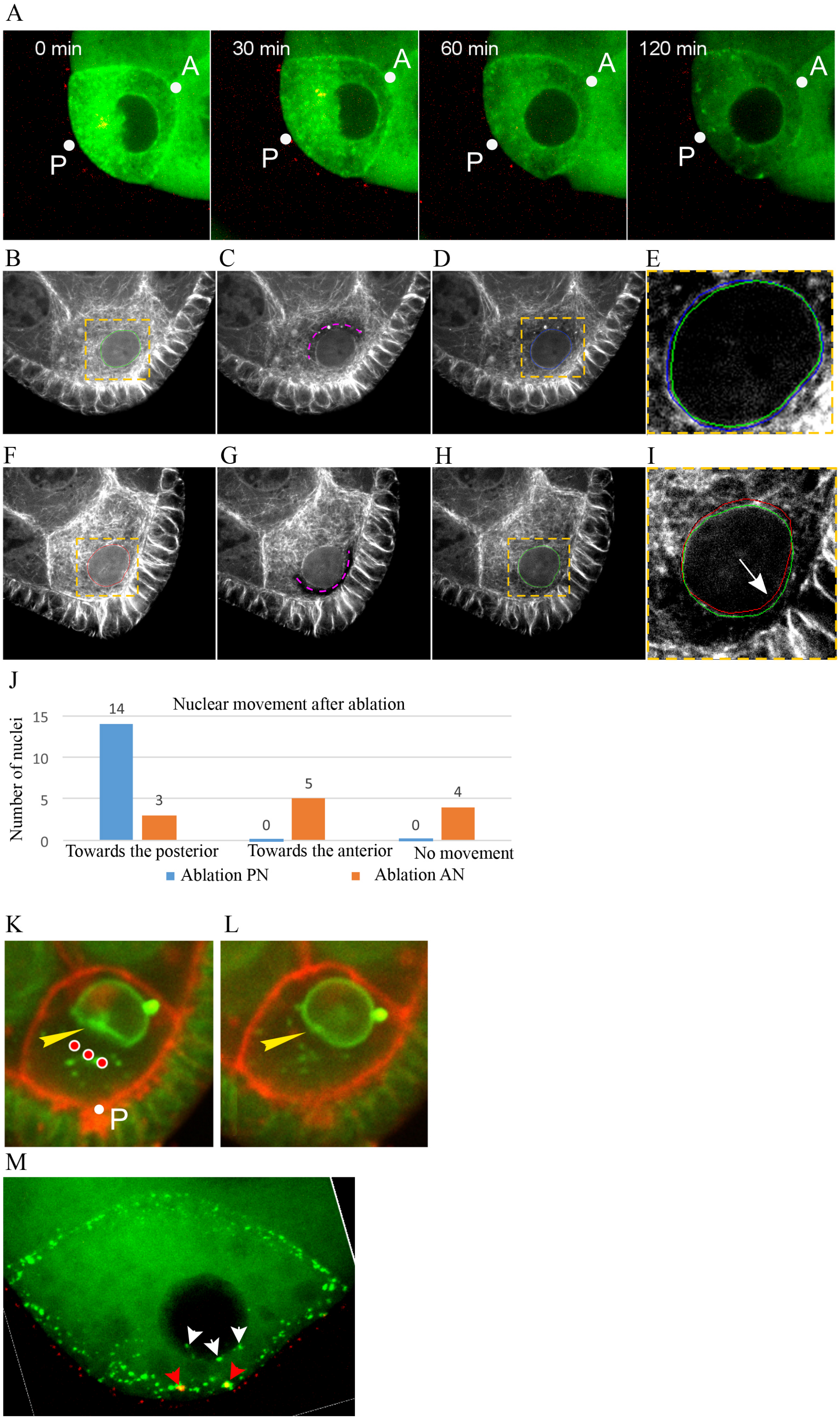
A
0 min
30 min
60 min
120 min
A
P
B
C
D
E
F
G
H
I
J
Nuclear movement after ablation
Number of nuclei
15
10
5
0
14
3
0
5
0
4
Towards the posterior
Towards the anterior
No movement
Ablation PN
Ablation AN
K
L
P
M

Supplementary Figure 2: MT forces applied on the nucleus

(A) Relaxation of the nuclear envelope indentation after MT depolymerisation by colcemid. Selected frames are displayed, respectively at the onset of drug incubation, 30, 60, and 120 min after drug incubation. EB1-GFP (green) and Asl-tdTomato (red). (B) Initial state of the oocyte before ablation. A close-up of the boxed region is shown in (E). (C) Immediately after nano-ablation along the pink dotted line. (D) 100s after ablation. The white dots correspond to residual aggregates from the ablation. The nucleus is circled in blue. (E) Overlay between images before and after ablation. The nuclear area increases slightly but shows no displacement. (F-I) Nucleus behaviour after nano-ablation between the nucleus and the posterior of the oocyte. (F) Initial state of the oocyte before ablation. The nucleus is circled in red. A close-up of the boxed region is shown in (I). (G) Just after ablation along the pink dotted line. (H) 100s after ablation. The nucleus is circled in green. (I) Overlay between images before and after ablation. The white arrow represents the vector of nuclear displacement towards the posterior, following ablation. (J) Histogram showing the nuclear shift after ablation between the nucleus and the posterior of the oocyte (PN) in blue (n=14) or between the nucleus and the anterior margin of the oocyte (AN) orange (n=12). (K, L) Relaxation of the nuclear envelope indentation after two photons-mediated nano-ablation posterior to the nucleus during migration. (K) Before ablation, the position of the ablation area is marked by the red dots. (L) After ablation, the indentation of the nuclear envelope is significantly relaxed. Fs(2)Ket-GFP (green) and $PH^{PLC-\partial 1}$-RFP (red). (M) MT nucleation sites highlighted with EB1-GFP are visualized as asters in the presence of colcemid that prevents further MT polymerization. The white arrows indicate MT nucleation at the nuclear envelope. The red arrows indicate MT nucleation at the centrosomes labelled with Asl-tdTomato (red).

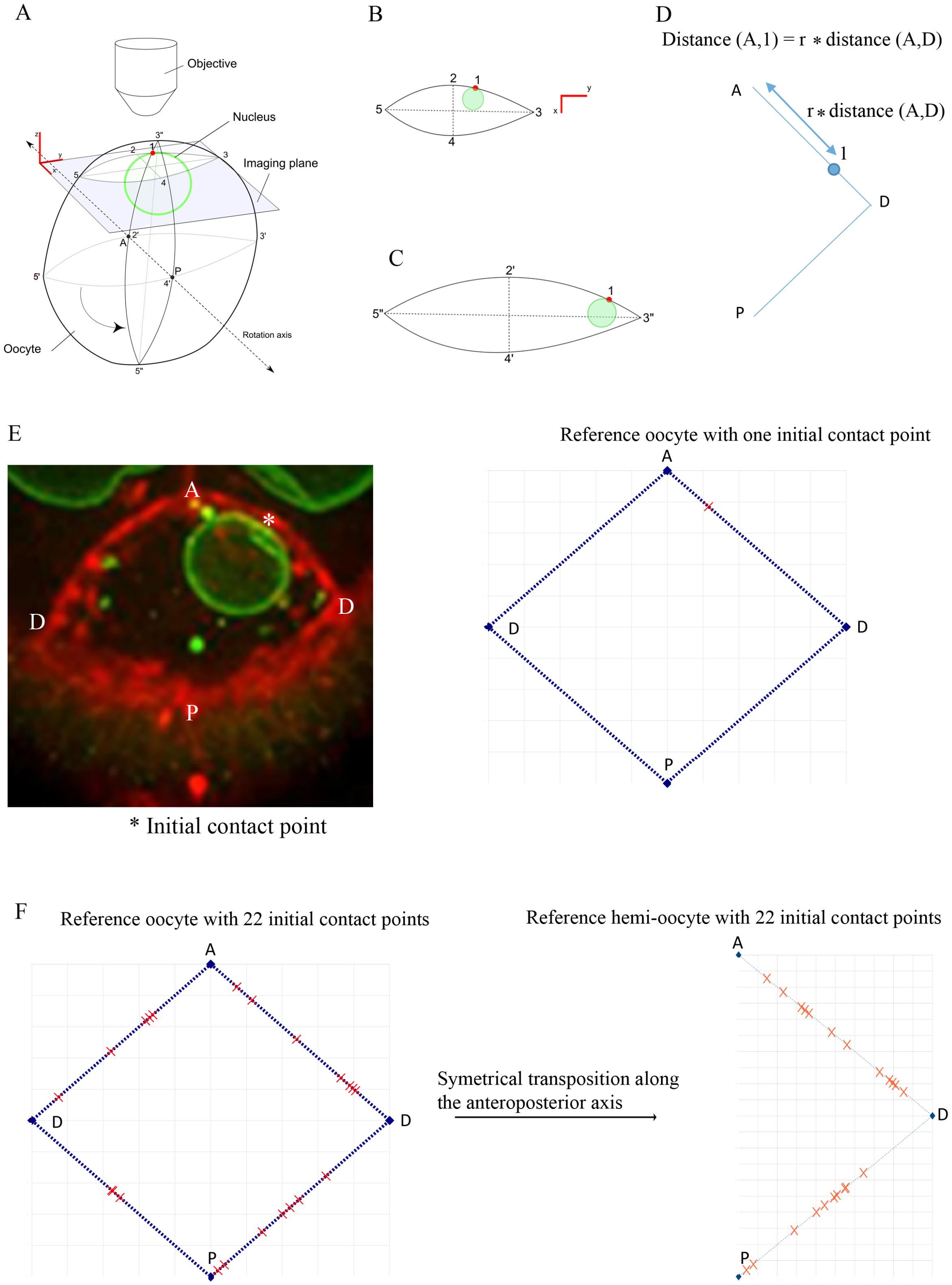
A
Objective
Nucleus
Imaging plane
Oocyte
Rotation axis
B
C
D
Distance (A,1) = r ∗ distance (A,D)
r ∗ distance (A,D)
E
* Initial contact point
Reference oocyte with one initial contact point
F
Reference oocyte with 22 initial contact points
Symetrical transposition along
the anteroposterior axis
Reference hemi-oocyte with 22 initial contact points

Supplementary Figure 3: Quantitative analysis of nuclear migrations in 3D.

(A, B, C) Geometrical visualization of the oocyte equatorial plane corresponding to the confocal acquisition plane in which the nucleus contacts the plasma membrane. (A) Global schematic representation of an oocyte in which the nucleus (green circle) contacts the APM at point 1 (red dot). A and P mark the anterior and posterior-most of the oocyte respectively. The xy plane (blue), orthogonal to the objective, is moving along the z-axis with a stepsize between 0.5 and 2 μm to cover the whole depth of the oocyte. The plane (2, 3, 4, 5) represents a confocal section where nucleus-APM contact can be observed, but this plane is not equatorial with regards to the oocyte. (2', 3', 4', 5') is the only equatorial plane that also corresponds to a confocal imaging plane orthogonal to the optical axis. Conversely (2', 3", 4', 5") is the only equatorial plane that comprises the nucleus-APM contact point (point 1, red dot) but it does not correspond to a confocal section. For each migration, a 3D analysis of the oocyte and determination of the relevant equatorial plane (2', 3", 4', 5") were achieved to position the contact point to the APM or the LPM in a normalized equatorial plane, in order to compare initial contact points for all migrations. (B) Initial contact point in its confocal section (2, 3, 4, 5). (C) Initial contact point in its equatorial plane (2', 3", 4', 5") equatorial plan of (A) containing the contact point. (D) Measurement of the position of the nuclear initial contact points (1) on the plasma membrane in the equatorial plane (A, P, D) correspond to (2', 3", 4'). r corresponds to the ratio : d(2', 1)/d(2', 3"). (E) Example of nucleus contacting the APM and its corresponding reference oocyte graph. (A, P, D) correspond to (2', 3', 4', 5') in (C). A: Anterior corresponds to 2', P: posterior corresponds to 4', and D: Dorsal correspond either to 3" or 5". (F) The reference oocyte with the 22 initial contact points can be projected onto a reference hemi-oocyte graph due to the oocyte axial symmetry along its A-P axis.
